# Prof. Horatio R. Storer’s Lectures to Physicians

**Published:** 1867-08

**Authors:** 


### Prof. Horatio R. Storer's Lectures to Physicians.

It will be observed by notice in our advertisement sheet that Prof. Storer will deliver a second course of lectures to physicians, upon the Surgical Diseases of Women. His first effort appears to have been eminently successful, and we notice that the physicians who attended the course, were men of experience and attainment in the profession. Perhaps there is no better way of representing the sentiment of his first class than by republishing their own *resolutions*, which we find in the June number of the Boston Medical and Surgical Journal.

"At a meeting of the physicians in attendance upon Prof. H. R. Storer's course of lectures on the Surgical Diseases of Woman, just delivered at Hotel Pelham in Boston, the following preamble and resolutions were adopted:

WHEREAS, We, the attendants upon Prof. H. R. Storer's first private course of lectures on the Surgical Diseases of Women, being regular practising Physicians and Surgeons, have long experienced the disadvantages arising from the very imperfect manner in which these subjects have been treated in our text-books, and by the professors in our colleges; many of the most important diseases and operations being entirely ignored, by men who think deeply and reason candidly in all other matters pertaining to medicine and surgery; and whereas, we cannot but feel that this class of diseases is the most important, believing it to be the cause of more suffering than any other, therefore,

*Resolved*, That we tender to Dr. Storer, our sincere gratitude for taking the advanced step which he has, thereby giving us, as we hope he will hereafter give others, the opportunity of hearing these subjects discussed thoroughly and impartially.

*Resolved*, That a copy of these resolutions be presented to Prof. Storer, and sent to the Medical and Surgical Journal and the New York Medical Record for publication. (Signed,)

CHAS. M. CARLTON, Norwich, Conn.

DANIEL MANN, Pelham, N. H.

E. G. BULLARD, Blackstone, Mass.

J. A. McDONOUGH, Boston, Mass.

M. C. TALBOT, Warren, Penn.

H. GEROULD, Erie, Penn.

E. F. UPHAM, W. Randolph, Vt.

G. J. ARNOLD, Roxbury, Mass.

W. A. I. CASE, Hamilton, C. W.

W. L. WELLS, Howell, Mich.

*Boston, June* 15, 1867.

Through the politeness of our friend, Dr. Lewis Krombein of this city, we are in receipt of a specimen of *Ferri Carbonas Effervescence*, prepared by Dr. F. Weidler of Cincinnati. It appears to be a palatable preparation of iron, and may prove acceptable when iron in other forms is not well borne.

